# Association Between Chronic Inflammation, Bone Density, and Muscle in Peripubertal Adolescents With and Without HIV in Zimbabwe: A Cross-sectional Study

**DOI:** 10.1093/ofid/ofag322

**Published:** 2026-05-22

**Authors:** Lisha Jeena, Rashida A Ferrand, Victoria Simms, Cynthia Kahari, Tsitsi Bandason, Ruramayi Rukuni, Andrea M Rehman, Sarah Rowland-Jones, Celia L Gregson, Anthony Y Y Hsieh

**Affiliations:** Oxford Centre for Immuno-Oncology, Nuffield Department of Medicine, University of Oxford, Oxford, United Kingdom; Clinical Research Department, Faculty of Infectious and Tropical Diseases, London School of Hygiene and Tropical Medicine, London, United Kingdom; The Health Research Unit Zimbabwe, Biomedical Research and Training Institute, Harare, Zimbabwe; The Health Research Unit Zimbabwe, Biomedical Research and Training Institute, Harare, Zimbabwe; Medical Research Council International Statistics and Epidemiology Group, Department of Infectious Disease Epidemiology, Faculty of Epidemiology and Population Health, London School of Hygiene and Tropical Medicine, London, United Kingdom; The Health Research Unit Zimbabwe, Biomedical Research and Training Institute, Harare, Zimbabwe; Medical Research Council International Statistics and Epidemiology Group, Department of Infectious Disease Epidemiology, Faculty of Epidemiology and Population Health, London School of Hygiene and Tropical Medicine, London, United Kingdom; The Health Research Unit Zimbabwe, Biomedical Research and Training Institute, Harare, Zimbabwe; Clinical Research Department, Faculty of Infectious and Tropical Diseases, London School of Hygiene and Tropical Medicine, London, United Kingdom; The Health Research Unit Zimbabwe, Biomedical Research and Training Institute, Harare, Zimbabwe; Medical Research Council International Statistics and Epidemiology Group, Department of Infectious Disease Epidemiology, Faculty of Epidemiology and Population Health, London School of Hygiene and Tropical Medicine, London, United Kingdom; Oxford Centre for Immuno-Oncology, Nuffield Department of Medicine, University of Oxford, Oxford, United Kingdom; The Health Research Unit Zimbabwe, Biomedical Research and Training Institute, Harare, Zimbabwe; Musculoskeletal Research Unit, Translational Health Sciences, Bristol Medical School, University of Bristol, Bristol, United Kingdom; Oxford Centre for Immuno-Oncology, Nuffield Department of Medicine, University of Oxford, Oxford, United Kingdom

**Keywords:** adolescence, bone, HIV, inflammation, muscle

## Abstract

**Background:**

We aimed to investigate the effect of human immunodeficiency virus (HIV-1)–associated inflammation on bone and muscle outcomes in adolescents with HIV (AWH) and adolescents without HIV (AWOH) and, among AWH, the impact of HIV viral load and antiretroviral treatment (ART) duration.

**Methods:**

AWH (on ART ≥2 years) and AWOH aged 8–16 years were enrolled. Luminex technology quantified the biomarkers C-reactive protein (CRP), soluble CD14 (sCD14), TNF-α, IL-6, IL-17, IL-18, IL-10, and IFN-γ—chosen based on their relevance to HIV, bone, and muscle. Factorial analysis of mixed data reduced measurements into components. Sex-stratified adjusted linear regression assessed associations between HIV status and biomarker components, HIV-related factors (viral load and ART duration) and components, and components and bone/muscle outcomes. Where associations were found, individual biomarkers were investigated. Outcomes included height-adjusted total-body-less-head bone mineral content for lean mass (TBLH-BMC^LBM^) and size-adjusted lumbar-spine bone mineral apparent density (LS-BMAD) Z-scores, TBLH-lean mass, grip strength, and lower limb power.

**Results:**

There were 270 AWH (mean age, 12.6 [SD, 2.5] years; 49.6% female) and 287 AWOH (mean age, 12.7 [SD, 2.5] years; 50.4% female). Increased component 1 driven by IL-18, CRP, sCD14, and TNF-α was associated with HIV status. Unexpectedly, among girls with HIV, higher component 1 was associated with greater LS-BMAD Z-score (adjusted mean difference, β-coefficient 0.15 [95% CI, .01–.29]; *P* = .036); this was driven by IL-18 that was positively associated with LS-BMAD (0.90 [95% CI, .26–1.55]; *P* = .013) and TBLH-BMC^LBM^ (0.72 [95% CI, .14–1.30]; *P* = .043) Z-scores. Biomarkers were not associated with HIV viral load, ART duration, or any muscle outcomes.

**Conclusions:**

Biomarkers (IL-18, CRP, sCD14, TNF-α) were increased in AWH and may be linked to higher bone density in girls with HIV. IL-18 plays an important role on bone that has not been previously described and warrants further investigation.

## Teaser

Increased biomarkers were shown in peripubertal children who acquire HIV at birth. Unexpectedly, this increased inflammation was associated with increased bone density in girls living with HIV, driven by a novel biomarker—IL-18.

Approximately 90% of children and adolescents (<15 years of age) with human immunodeficiency virus (HIV-1) (CWH and AWH, respectively) live in sub-Saharan Africa [1]. Expanded access to antiretroviral treatment (ART) has reduced human immunodeficiency virus (HIV)–related childhood mortality [2–4], yet perinatally acquired HIV continues to cause significant morbidity through adolescence [3, 4]. Puberty is critical for bone and muscle development, which HIV may disrupt [5]. Our group and others have shown impaired bone growth, low bone density, and reduced muscle function through puberty in AWH, even on ART with viral suppression [6–10]. Understanding the mechanisms could inform interventions to improve adolescent bone and muscle and reduce future risk of osteoporosis, sarcopenia, fractures, and falls [11].

Chronic inflammation persists in CWH and AWH despite ART and is associated with pulmonary, cardiovascular, and neurocognitive comorbidities [4], but its effects on bone density and muscle are less clear. During puberty, hormonal changes promote bone accrual by osteoblasts, while osteoclasts remodel and resorb bone [12, 13]. Cytokines that impair bone in inflammatory conditions such as rheumatoid arthritis are also elevated with HIV and may disrupt osteoblast and/or osteoclast activity [14–17]. In prepubertal South African CWH (mean age, 7 years), increased interleukin (IL)-6 and C-reactive protein (CRP) were negatively associated with bone formation at enrollment, tumor necrosis factor alpha (TNF-α) was positively associated with both bone formation and resorption at 2-year follow-up, and soluble CD14 (sCD14) was associated with impaired percent-change in bone [9]. In older CWH (mean age, 12 years) in the United States, interferon gamma (IFN-γ), TNF-α, sCD14, and IL-17 were associated with lower bone density in boys (Tanner stages III–IV), while increased IFN-γ, TNF-α, and sCD40 ligand were associated with higher bone density in girls (Tanner stage IV) [18]. This indicates potential sex-specific effects of HIV-associated inflammation, particularly relevant for peripubertal children, as girls typically enter puberty earlier and are at a higher Tanner stage at a given chronological age compared to boys [19].

Effects of HIV-associated inflammation on muscle are lesser studied. Among US adults with HIV (mean age, 36 years), higher IL-6, sCD14, CRP, and sCD163 were associated with poorer psoas muscle quality (measured by fatty infiltration on computed tomography), both before and 96 weeks after ART initiation [20]. Adults with HIV also experience earlier onset of sarcopenia [21]. HIV-associated inflammation may play a role by increasing protein degradation and muscle catabolism [22, 23].

In this study, we investigate the association between HIV and biomarkers, the effect of HIV-related factors (ART duration and viral load) on biomarkers, and the association between biomarkers and bone density, muscle mass, and strength in Zimbabwean peripubertal AWH on ART compared to adolescents without HIV (AWOH).

## MATERIALS AND METHODS

### Study Design and Participants

This cross-sectional study included AWH aged 8–16 years, on ART for ≥2 years, from 2 public hospitals (Parirenyatwa and Sally Mugabe) in Harare, Zimbabwe, and age- and sex-frequency-matched AWOH from nearby government schools. We enrolled participants in the IMVASK (Impact of Vertical HIV Infection on Child and Adolescent Skeletal Development) cohort between June 2018 and November 2019 [24]. The study team approached and recruited AWH sequentially during routine HIV clinic visits, and enrolled up to 5 participants daily. The study team approached AWOH from schools proportional to school size and using a random number sequence. We applied quota sampling by sex and age group (8–10, 11–13, 14–16 years) to ensure balanced recruitment (Supplementary Figure 1). We excluded adolescents who were acutely unwell or lived outside Harare [24].

### Questionnaire

Sociodemographic (age, sex, socioeconomic status [SES], orphanhood), lifestyle (physical activity, vitamin D and calcium intake, tuberculosis history), and HIV-specific data (age at diagnosis, age at ART initiation, tenofovir disoproxil fumarate [TDF] use, and ART duration as a percentage of life) were collected via researcher-administered questionnaires. Vitamin D intake was assessed using a locally validated tool based on consumption of animal-sourced foods (eggs, dairy, fish, meat, fortified oils, margarine, kapenta) [24, 25]. Physical activity was measured in MET-minutes (metabolic equivalent of task) using the International Physical Activity Questionnaire, validated in several countries, including South Africa [26].

### Clinical Examination

Standing height and weight were measured in duplicate using a Seca 213 stadiometer (to 0.1 cm) and Seca 875 scales (to 0.1 kg), respectively. A third measurement was taken if differences exceeded 0.5 cm or 0.5 kg; final values were the mean of 2 or 3 measurements. Height- and weight-for-age Z-scores were calculated using 1990 UK reference data due to lack of local standards [27]. Pubertal stage (Tanner I–V) was assessed based on testicular and pubic hair development in boys, and breast development, pubic hair, and menarche in girls [28, 29]. Discrepancies were resolved using breast or testicular staging.

### Blood Sample Collection

A fasting blood sample (up to 15 mL) was collected at enrollment. For AWH, CD4^+^ T-cell count and HIV viral load were measured using the Alere PIMA CD4 machine and the Cepheid GeneXpert platform, respectively. Participants without recent documented HIV-negative results provided assent (with parental/guardian consent) for rapid HIV testing before enrollment. Peripheral blood mononuclear cells and plasma were isolated the same day. Samples were stored in approximately 1-mL cryovials at −80°C and shipped in liquid nitrogen to the University of Oxford per the collaboration agreement [24].

### Measurement of Plasma Soluble Biomarkers

Samples were reorganized into a randomized order before processing. Eight plasma biomarkers (CRP, sCD14, TNF-α, IL-6, IL-17, IL-18, IL-10, IFN-γ) were selected based on prior literature on their role in HIV-associated inflammation and relevance to bone and muscle [15, 30]. Biomarkers were measured using Luminex bead arrays (BioRad200) per manufacturer protocol. Samples were thawed once, appropriately diluted, and run in duplicate. Analyte concentrations were calculated by interpolating the fluorescence values from a manufacturer-provided standard curve (Biotechne Luminex Multiplex Discover Assay, catalog number LXSAHM, R&D Systems). Raw fluorescence values were background-corrected by subtracting the blank, and final concentrations were adjusted for sample dilution. The final value for each sample was reported as the mean of the 2 technical replicates.

### Bone Density Measurement

Bone measurements included total-body-less-head bone mineral content for lean mass (TBLH-BMC^LBM^) and lumbar-spine bone mineral apparent density (LS-BMAD), assessed by dual X-ray absorptiometry (DXA) using a Hologic QDR-WI densitometer with Apex software V.4.5 (Hologic). Daily calibration used a manufacturer-provided spine phantom. Scan reproducibility was checked in a subgroup of participants (n = 30). LS-BMAD was calculated using the Carter method [31]. TBLH-BMC^LBM^ was derived using equations adjusting for log-transformed lean and fat mass, height, and age [32]. Both measures were adjusted for bone size [33]. Z-scores were generated using UK reference data from mainly White adolescents (aged 4–20 years), scanned between 1996 and 2012 on a Hologic system [32, 34].

### Grip Strength

Participants were seated with their forearm resting on the chair arm, elbow flexed at a right angle, and wrist in a neutral position. Using a Jamar hand-held dynamometer (handle position II), grip strength was measured 3 times per hand, alternating sides. The highest grip strength measurement from 6 measurements was used for analysis.

### Standing Long Jump (Lower Limb Power)

Participants stood on a hard surface with heels on the starting line and feet parallel. A research nurse provided instructions and demonstrated the task. Participants were asked to jump forward as far as possible, landing with both feet together without stepping forward. Three jumps were performed, each measured in centimeters using a measuring tape from the starting line to the heel closest to it. The longest distance was used for analysis.

### Body Composition (Lean Mass)

The same DXA scans that measured bone density also measured TBLH fat and fat-free (ie, lean) mass using a standard protocol on Apex version 4.5 software for scan acquisition analysis.

### Statistical Analysis

Demographic characteristics were compared by HIV status, stratified by sex, using independent sample *t*-tests to compare means and χ^2^ tests to compare proportions. SES category was based on the first component of a principal component analysis, split into tertiles. SES variables included number of people in the household; age of the household head; maternal and paternal educational attainment; household ownership; monthly income; access to electricity, water, and flush toilet; and household ownership of a refrigerator, bicycle, car, television, or radio.

Cytokine measurements were analyzed as either continuous or categorical (ie, detectable vs undetectable) variables based on the number of measurements that were within or below the limit of detection: IL-18, CRP, sCD14, and TNF-α were analyzed as continuous variables; IL-6, IL-10, IFN-γ, and IL-17 were analyzed as categorical variables.

Factorial analysis of mixed data, using the FactoMineR package, reduced dimensionality of the data into 5 components that explained 80% of the data variance. Components 1 and 2, with eigenvalues of >1, captured 44% of the variance and were used for analysis. Continuous variables were also binarized to check for consistency in the reduction of biomarkers into components.

Sex-stratified analysis was performed given known sex-specific differences in biomarkers, bone density, muscle mass, and strength during puberty [35–37]. First, linear regression, adjusting for a priori factors age (years) and pubertal stage (5 Tanner stages), assessed the association between HIV status and biomarker components. Potential confounding variables were identified and assessed using a directed acyclic graph to inform the necessary adjustments. In the event of an association being observed with a component, HIV-associated characteristics (ART duration [years] and HIV viral load [<50, 50–1000, >1000 copies/mL]) were investigated for their associations with that component stratified by sex, using linear regression adjusted for a priori factors age and pubertal stage.

Next, to investigate the relationship between biomarkers and bone density and muscle outcomes, components 1 and 2 were first correlated against bone density and muscle mass and strength (LS-BMAD, TBLH-BMC^LBM^, grip strength, standing long jump, and lean mass) using Pearson correlation, stratified by sex and HIV status. Then, linear regression analysis investigated the association between component 1 and 2 and bone density, muscle mass, and strength, stratified by HIV status and sex. We adjusted for a priori factors age, pubertal stage, and fat mass (kilograms) (calculated as the percentage of TBLH-fat relative to the total of TBLH-fat, lean mass, and bone mineral content) as these factors are described in the literature to be associated with both biomarkers and bone and muscle measures [7, 38–40].

Last, in the event of finding an association between any component and a bone density or muscle outcome among AWH, an exploratory analysis was performed to delineate if there is an association between any individual biomarker within that component and the same bone density or muscle outcome. In this exploratory analysis we adjusted for the same a priori factors age, pubertal stage, and fat mass, with Benjamini–Hochberg *P* value correction for multiple comparisons [41].

Missing data were imputed using multiple imputation by chained equations as data were assumed to be missing at random [42]. Imputed variables included bone density, muscle grip strength, long jump distance, lean mass, age, sex, pubertal stage, SES, orphanhood, physical activity, vitamin D intake, and tuberculosis history. Adolescents with and without missing data and those with and without a blood plasma sample were compared.

Data were first processed in MS Excel software, and analyses were performed using RStudio (version 4.1.2) software.

### Patient Consent Statement

Parents or guardians provided written informed consent, and adolescents gave assent. The study was approved by the Medical Research Council of Zimbabwe (MRCZ/A/2297), Parirenyatwa Hospital and College of Health Sciences Joint Research Ethics Committee (JREC/11/18), Harare Central Hospital Ethics Committee (HCHEC 170118/04), Biomedical Research and Training Institute Institutional Review Board (AP145/2018), and London School of Hygiene and Tropical Medicine Ethics Committee (15333).

## RESULTS

### Study Population

Of 609 adolescents enrolled in the IMVASK study, 557 (91.5%) had a plasma sample available: 270 AWH (mean age, 12.6 [SD, 2.5] years; 49.6% girls) and 287 AWOH (mean age, 12.7 [SD, 2.5] years; 50.4% girls) (Table 1 and Supplementary Figure 1). AWH were more often at earlier Tanner stages than AWOH. A higher proportion of girls with HIV were in Tanner stages IV–V than boys with HIV (31 [23.13%] vs 22 [16.18%], respectively). Orphanhood and prior tuberculosis were more common among AWH. Girls with HIV were more likely to be of lower SES, while no difference was observed among boys. AWH had lower physical activity levels, shorter stature, and lower weight than AWOH. Girls with HIV had lower fat mass, lower lean mass, lower TBLH-BMC^LBM^, and lower grip strength, whereas boys with HIV had lower TBLH-BMC^LBM^, lower grip strength, lower lean mass, and shorter jump distance, compared to AWOH (Table 1).

**Table 1.** Baseline Characteristics of Study Participants

Characteristic	Girls (n = 282)			Boys (n = 275)		
	HIV Negative (n = 148)	HIV Positive (n = 134)	*P* Value	HIV Negative (n = 139)	HIV Positive (n = 136)	*P* Value
Sociodemographic characteristics						
Age, y, mean (SD)	12.6 (2.5)	12.4 (2.6)	.527	12.5 (2.5)	12.5 (2.5)	.839
Socioeconomic status, No. (%)						
Tertile 1 (low)	49 (33.1)	55 (41.0)	.007	38 (27.3)	49 (36.0)	.289
Tertile 2 (middle)	40 (27.0)	49 (36.6)		49 (35.3)	44 (32.4)	
Tertile 3 (high)	59 (39.9)	30 (22.4)		52 (37.4)	43 (31.6)	
Orphanhood (one or both parents), No. (%)^a^	8 (5.4)	60 (44.8)	<.001	11 (7.9)	53 (39.0)	<.001
Pubertal stage^b^, No. (%)						
Tanner I	24 (16.2)	53 (41.1)	<.001	40 (28.8)	52 (40.9)	.036
Tanner II	34 (23.0)	19 (14.7)		31 (22.3)	33 (26.90)	
Tanner III	28 (18.9)	26 (20.2)		23 (16.5)	20 (15.7)	
Tanner IV	49 (33.1)	24 (18.6)		41 (29.5)	18 (14.2)	
Tanner V	13 (8.8)	7 (5.4)		4 (2.9)	4 (3.1)	
Lifestyle factors						
Physical activity level, No. (%)						
Low (<600 MET-min/week)	62 (41.9)	68 (50.7)	.094	46 (33.1)	61 (44.9)	.067
Moderate (600–3000 MET-min/week)	37 (25.0)	37 (27.6)		47 (33.8)	31 (22.8)	
High (>3000 MET-min/week)	49 (33.1)	29 (21.6)		46 (33.1)	44 (32.4)	
Daily dietary vitamin D, No. (%)						
Very low (<4.0 μg/day)	6 (4.1)	8 (6.0)	.220	7 (5.0)	6 (4.4)	.497
Low (4.0–5.99 μg/day)	79 (53.4)	82 (61.2)		74 (53.2)	82 (60.3)	
Moderate (6.0–8.0 μg/day)	63 (42.6)	44 (32.9)		58 (41.7)	48 (35.3)	
History of tuberculosis, No. (%)^c^	0 (0.0)	18 (13.4)	<.001	2 (1.4)	26 (19.1)	<.001
HIV characteristics						
Age at HIV diagnosis, y, median (IQR)	…	3.13 (1.20–6.20)		…	2.99 (1.23–6.0)	
Age at ART initiation, y, median (IQR)	…	3.84 (1.85–7.54)		…	3.93 (1.97–6.77)	
% of life on ART, mean (SD)	…	64.51 (22.4)		…	67.73 (22.3)	
ART duration, y, mean (SD)	…	7.73 (2.53)		…	7.89 (2.78)	
TDF use (at baseline visit), No. (%)	…	46 (34.3)		…	44 (32.4)	
Viral load^d^, No. (%)						
<50 copies/mL	…	82 (61.7)		…	71 (52.6)	
50–1000 copies/mL	…	24 (18.0)		…	35 (26.0)	
>1000 copies/mL	…	27 (20.3)		…	29 (21.5)	
CD4^+^ T-cell count ≥500 cells/µL^e^, No. (%)	…	103 (76.9)		…	102 (75.0)	
Anthropometry						
Height, cm, mean (SD)	147.1 (12.1)	140.5 (13.0)	<.001	148.3 (14.7)	139.6 (12.2)	<.001
Weight, kg, mean (SD)	41.7 (12.7)	35.5 (10.7)	<.001	38.7 (11.2)	32.7 (7.6)	<.001
Fat mass, kg, mean (SD)	29.80 (6.5)	25.1 (5.4)	<.001	20.7 (5.0)	19.7 (3.9)	.081
Musculoskeletal measurements						
Bone density outcomes						
LS-BMAD, g/cm^3^, mean (SD)^f^	0.23 (0.0)	0.22 (0.0)	.065	0.19 (0.0)	0.19 (0.0)	.075
LS-BMAD Z-score, mean (SD)	0.11 (1.1)	−0.12 (1.3)	.131	−0.64 (1.2)	−0.88 (1.4)	.142
TBLH-BMC^LBM^, g, mean (SD)^g^	1079.1 (341.5)	928.4 (292.2)	<.001	1035.1 (359.1)	851.3 (246.6)	<.001
TBLH-BMC^LBM^ Z-score, mean (SD)	−0.28 (1.1)	−0.56 (1.1)	.036	−0.59 (0.9)	−0.68 (1.0)	.428
Muscle outcomes						
Grip strength, kg, mean (SD)	24.2 (7.9)	19.9 (7.1)	<.001	27.1 (10.3)	20.3 (7.3)	<.001
Jump power, cm, mean (SD)^h^	123.3 (18.9)	121.0 (21.7)	.345	143.5 (25.7)	128.1 (25.1)	<.001
TBLH-lean mass, kg, mean (SD)	29.2 (7.3)	26.0 (6.8)	<.001	30.4 (9.23)	25.9 (6.6)	<.001

Abbreviations: ART, antiretroviral treatment; HIV, human immunodeficiency virus; LS-BMAD, lumbar-spine bone mineral apparent density; MET, metabolic equivalent of task; SD, standard deviation; TBLH-BMC^LBM^, total-body-less-head bone mineral content for lean body mass; TDF, tenofovir disoproxil fumarate.

Missing data points:

^a^Orphanhood (1 boy without HIV, 7 boys with HIV; 2 girls without HIV; 5 girls with HIV).

^b^Pubertal stage (Tanner) (9 boys with HIV, 5 girls with HIV).

^c^History of tuberculosis (1 girl without HIV, 1 girl with HIV).

^d^Viral load (1 boy, 1 girl).

^e^CD4^+^ T-cell count (3 boys, 9 girls).

^f^LS-BMAD (6 boys, 10 girls).

^g^TBLH-BMC^LBM^ (7 boys, 10 girls).

^h^Jump power (2 boys, 3 girls).

Overall, 90.7% had complete data; missingness was highest for orphanhood (2.7%) and TBLH-BMC^LBM^ (3.1%) (Supplementary Materials 1, Supplementary Tables 1 and 2). Biomarker distribution is shown in Supplementary Table 3. Component 1 was primarily loaded by IL-18, CRP, sCD14, and TNF-α, while component 2 was loaded by IL-6, IL-10, IL-17, and IFN-γ (Supplementary Materials 1, Supplementary Figure 2*A* and 2*B*).

### HIV Characteristics

Among the 270 AWH, median age of HIV diagnosis was 3.0 (interquartile range [IQR], 1.2–6.0) years, with a median age at ART initiation of 3.8 (IQR, 1.9–7.0) years (Table 1). The mean percentage of life on ART was 64.84% (SD, 22.32%) overall; mean ART duration was 7.73 (SD, 2.53) and 7.89 (SD, 2.78) years among girls and boys, respectively. Forty-six (34.4%) girls and 44 (32.4%) boys were receiving TDF-based ART. There were 31 (23.1%) girls and 34 (25.0%) boys with CD4^+^ T-cell count <500 cells/µL, and 27 (20.3%) girls and 29 (21.5%) boys with HIV viral load ≥1000 copies/mL (Table 1).

### Association Between Biomarkers and HIV

Component 1 was strongly associated with HIV in both sexes, both before and after adjusting for age and pubertal stage (adjusted mean difference, 0.62 [95% confidence interval {CI}, .26–.99], *P* = .001 among girls and 0.75 [95% CI, .39–1.10], *P* < .001 among boys) (Table 2). The effect sizes before and after adjustment were similar. Among boys, HIV was associated with lower component 2 in univariable analysis (−0.29 [95% CI, −.57 to −.00]; *P* = .048), which was attenuated after adjusting for age and pubertal stage (−0.22 [95% CI, −.52 to .07]; *P* = .132) (Table 2).

**Table 2.** Linear Regression Coefficients (With 95% Confidence Interval) for the Associations Between HIV and the 2 Biomarker Components

Component	Unadjusted β Coeff (95% CI)	*P* Value	Adjusted β Coeff (95% CI)^a^	*P* Value
Girls (n = 282)				
Component 1	0.79 (.45–1.12)	<.001	0.62 (.26–.99)	.001
Component 2	0.01 (−.28 to .29)	.970	0.03 (−.28 to .34)	.846
Boys (n = 275)				
Component 1	0.76 (.41–1.10)	<.001	0.75 (.39–1.10)	<.001
Component 2	−0.29 (−.57 to −.00)	.048	−0.22 (−.52 to .07)	.132

β coefficient is the increase or decrease in component 1 or 2 biomarkers by living with HIV.

Abbreviations: CI, confidence interval; Coeff, coefficient.

^a^Adjusted for age (years) and pubertal stage (5 Tanner stages).

### Factors Associated With Biomarkers Among AWH

HIV characteristics, viral load, and ART duration were not associated with component 1 (Supplementary Table 4). HIV characteristics were not investigated for component 2 because component 2 was not associated with HIV status.

### Associations Between Biomarkers and Bone Density

**Figure 1:**
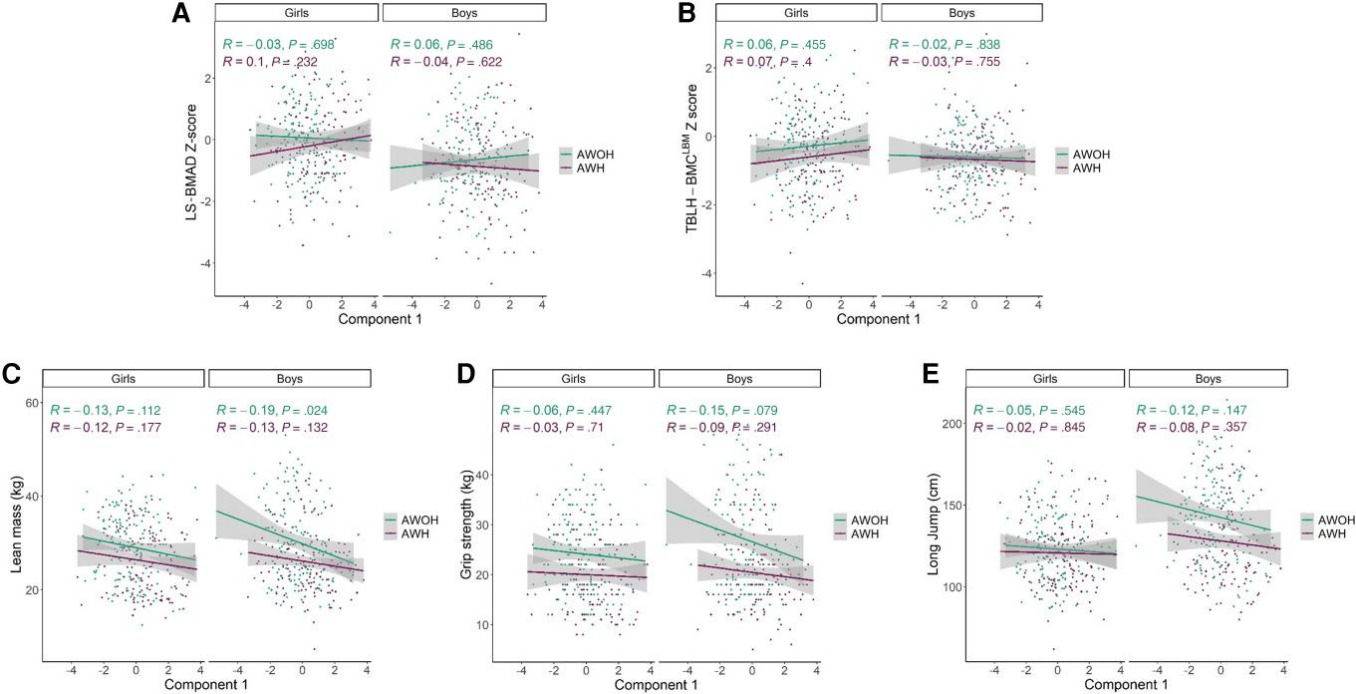
Correlations between component 1 biomarkers and musculoskeletal outcomes: LS-BMAD (*A*), TBLH-BMC^LBM^ (*B*), lean mass (*C*), grip strength (*D*), and long jump (*E*). Unadjusted data are shown. Pearson correlations (*R*) shown with *P* values. Error bars (in gray) indicate 95% confidence intervals. Data presented in teal represent AWOH and purple represents AWH. Abbreviations: AWH, adolescents with human immunodeficiency virus; AWOH, adolescents without human immunodeficiency virus; LS-BMAD, lumbar-spine bone mineral apparent density; TBLH-BMC^LBM^, total-body-less-head bone mineral content for lean body mass.

There was no evidence of a univariable association between component 1 (primarily IL-18, CRP, sCD14, TNF-α) or component 2 (primarily IL-6, IL-10, IL-17, IFN-γ,) and bone density (Figure 1*A* and 1*B*, Table 3, Supplementary Figure 3*A* and 3*B*). However, after adjusting for age, pubertal stage, and fat mass, and only in girls with HIV, a unit increase of component 1 was associated with greater LS-BMAD Z-score (0.15 [95% CI, .01–.29]; *P* = .036); this was not observed for TBLH-BMC^LBM^ Z-score (Table 3). No univariable nor multivariable associations were found between component 2 and either LS-BMAD or TBLH-BMC^LBM^ Z-scores in either girls or boys with HIV (Table 3). In AWOH, component 1 was not associated with either LS-BMAD or TBLH-BMC^LBM^ Z-score, in either univariable or multivariable analysis. Among girls without HIV, per-unit increase in component 2 was univariably associated with greater LS-BMAD and TBLH-BMC^LBM^ Z-score (0.15 [95% CI, .00–.31], *P* = .050 and 0.15 (0.00 [95% CI, .00–.29], *P* = .043), respectively), but not in multivariable analysis (Supplementary Table 5). Among boys without HIV, there was no univariable association between component 2 and either bone outcome. Higher component 2 was associated with lower TBLH-BMC^LBM^ Z-score in multivariable analysis adjusted for age, pubertal stage, and fat mass (−0.13 [95% CI, −.25 to .02]; *P* = .026) but not with LS-BMAD Z-score (Supplementary Table 5).

**Table 3.** Linear Regression Coefficients (With 95% Confidence Interval) for the Association Between Biomarker Components and Bone Outcomes Among Adolescents With HIV

Component	LS-BMAD Z-Score				TBLH-BMC^LBM^ Z-Score			
	Unadjusted β Coeff (95% CI)	*P* Value	Adjusted β Coeff (95% CI)^a^	*P* Value	Unadjusted β Coeff (95% CI)	*P* Value	Adjusted β Coeff (95% CI)^a^	*P* Value
Girls (n = 134)								
Component 1	0.09 (−.06 to .24)	.232	0.15 (.01–.29)	.036	0.06 (−.07 to .19)	.400	0.11 (−.02 to .23)	.105
Component 2	0.03 (−.16 to .21)	.789	0.05 (−.13 to .22)	.608	−0.01 (−.17 to .15)	.933	−0.02 (−.18 to .13)	.767
Boys (n = 136)								
Component 1	−0.04 (−.19 to .12)	.622	−0.07 (−.21 to .06)	.287	−0.02 (−.13 to .09)	.755	−0.03 (−.12 to .09)	.636
Component 2	0.01 (−.20 to .22)	.901	−0.01 (−.21 to .18)	.887	0.06 (−.09 to .21)	.434	0.05 (−.11 to .21)	.540

β coefficient is the increase or decrease in component 1 or 2 biomarkers per 1-unit increase in the bone density Z-score.

Abbreviations: CI, confidence interval; Coeff, coefficient; LS-BMAD, lumbar-spine bone mineral apparent density; TBLH-BMC^LBM^, total-body-less-head bone mineral content for lean body mass.

^a^Adjusted for age (years), pubertal stage (5 Tanner stages), and fat mass (kilograms).

When further separated by pubertal stage, there was no univariable association between either component 1 or 2 and either bone outcome. Multivariable analysis adjusting for age, pubertal stage, and fat mass showed an association between higher component 1 and greater LS-BMAD Z-score in girls with HIV in Tanner stages III–V (0.21 [95% CI, .03–.40]; *P* = .024) and between higher component 2 and greater TBLH-BMC^LBM^ in boys with HIV in Tanner stages III–V (0.31 [95% CI, .04–.58]; *P* = .024) (Supplementary Table 6).

Since component 1 was associated with bone outcomes, we continued to exploratory analysis of the individual biomarkers that primarily contributed to it. Among girls with HIV, IL-18 was strongly associated with both LS-BMAD and TBLH-BMC^LBM^ Z-score after adjusting for age, pubertal stage, and fat mass (0.90 [95% CI, .26–1.55], *P* = .013 and 0.71 [95% CI, .14–1.30], *P* = .043, respectively) (Table 4). There was no univariable association between component 1 cytokines and bone outcomes; the effect sizes increased with multivariable analysis. There was no association between IL-18, CRP, sCD14, or TNF-α and bone density among boys with HIV.

**Table 4:** Linear Regression Coefficients (With 95% Confidence Interval) for the Association of Individual Biomarkers in Component 1 and Bone Density Among Adolescents With HIV

Biomarker	LS-BMAD Z-Score					TBLH-BMC^LBM^ Z-Score				
	Unadjusted β Coeff (95% CI)	*P* Value	Adjusted^a^ β Coeff (95% CI)	*P* Value	BH-Corrected *P* Value	Unadjusted β Coeff (95% CI)	*P* Value	Adjusted^a^ β Coeff (95% CI)	*P* Value	BH-Corrected *P* Value
Girls (n = 134)										
Log_10_ IL-18	0.59 (−.10 to 1.28)	.093	0.90 (.26–1.55)	.006	.013	0.45 (−.15 to 1.05)	.137	0.71 (.14–1.30)	.016	.043
Log_10_ CRP	0.03 (−.25 to .30)	.851	0.05 (−.21 to .32)	.698	.698	0.06 (−.18 to .30)	.611	0.10 (−.14 to .34)	.400	.457
Log_10_ sCD14	0.50 (−.39 to 1.39)	.269	0.72 (−.13 to 1.56)	.097	.130	0.46 (−.42 to 1.13)	.362	0.46 (−.30 to 1.22)	.233	.312
Log_10_ TNF-α	0.16 (−.28 to .61)	.468	0.26 (−.16 to .68)	.222	.254	−0.11 (−.50 to .28)	.580	−0.02 (−.40 to .36)	.916	.917
Boys (n = 136)										
Log_10_ IL-18	−0.35 (−1.13 to .43)	.372	−0.40 (−1.08 to .30)	.264	.352	−0.27 (−.83 to .29)	.349	−0.30 (−.88 to .28)	.310	.721
Log_10_ CRP	0.02 (−.28 to .32)	.894	−0.11 (−.38 to .16)	.430	.491	0.02 (−.20 to .24)	.842	−0.01 (−.24 to .22)	.934	.934
Log_10_ sCD14	0.00 (−.80 to .81)	.994	−0.12 (−.83 to .59)	.737	.736	0.08 (−.50 to .66)	.781	0.04 (−.56 to .63)	.901	.901
Log_10_ TNF-α	−0.55 (−1.17 to .07)	.080	−0.54 (−1.10 to .02)	.058	.154	−0.14 (−.59 to .31)	.533	−0.17 (−.64 to .31)	.487	.671

β coefficient is the increase or decrease in biomarker per 1-unit increase in the bone density Z-score.

Abbreviations: BH, Benjamini–Hochberg; CI, confidence interval; Coeff, coefficient; CRP, C-reactive protein; IL, interleukin; LS-BMAD, lumbar-spine bone mineral apparent density; TBLH-BMC^LBM^, total-body-less-head bone mineral content for lean body mass; sCD14, soluble CD14; TNF-α, tumor necrosis factor alpha.

^a^Adjusted for age (years), pubertal stage (5 Tanner stages), and fat mass (kilograms).

### Association Between Biomarkers and Muscle Mass and Strength

In AWH, component 1 was not associated with lean mass, grip strength, or long jump, in either univariable or in multivariable analysis adjusting for age, pubertal stage, and fat mass (Figure 1*C*–*E*, Supplementary Table 7). Higher component 2 was associated with greater grip strength among boys with HIV in univariable analysis (1.23 [95% CI, .15–2.30]; *P* = .025), but this was attenuated after adjustment (Supplementary Table 7, Supplementary Figure 3*D*). No other associations were observed between component 2 and muscle outcomes (Supplementary Table 7).

In AWOH, higher component 1 was univariably associated with lower lean mass among boys (−1.32 [95% CI, −2.46 to −.17]; *P* = .025) (Figure 1*C*, Supplementary Table 8), and higher component 2 was univariably associated with greater grip strength among girls without HIV (1.09 [95% CI, .03–2.15]; *P* = .043). Both associations were fully attenuated after adjusting for age, pubertal stage, and fat mass (Supplementary Figure 3*D*, Supplementary Table 8).

## DISCUSSION

In this study, component 1, primarily a group of proinflammatory biomarkers (IL-18, CRP, sCD14, TNF-α), was associated with HIV. This finding aligns with evidence that chronic inflammation persists in people with HIV despite viral suppression on ART [4, 43]. Unexpectedly, component 1 was positively associated with bone density in girls with HIV but not their HIV-negative peers, nor among boys. The difference by sex may relate to earlier onset of puberty in girls compared to boys, such that more girls were experiencing accelerated growth at the point of this study. Sex and growth hormones may exert an overall growth-promoting effect that favors bone formation over resorption, potentially outweighing the effects of underlying inflammation during puberty [44, 45]. Supporting this hypothesis, we observed a positive association between biomarkers and bone density at later pubertal stages in both sexes with HIV. Previous studies report negative associations between biomarkers and bone in early puberty and positive associations in late puberty, supporting sex- and Tanner stage–specific effects [9].

Exploratory analysis into component 1 found that IL-18 was positively associated with bone density Z-scores in girls with HIV, despite its usual proinflammatory role [46, 47]. This may be supported by in vitro evidence that IL-18 inhibits osteoclast differentiation through a synergistic effect with IL-12 to increase IFN-γ production; by stimulating granulocyte-macrophage colony-stimulating factor production by T cells, independent of IFN-γ; or by mediating myeloid apoptosis via Fas/Fas-ligand [48–50]. No direct pathway linking IL-18 to bone metabolism in vivo has been established. In HIV-infected cells in vitro, elevated IL-18 upregulates CXCR4 and TRAIL expression, promoting cell infection and apoptosis [51, 52]. Our findings, therefore, support a pleiotropic effect of IL-18 that is dependent on dose, or cell type, and one that may differ across body tissues or life stages.

While there are limited clinical data on the role of IL-18 during puberty, findings in postmenopausal women show high serum IL-18 levels with osteoporosis or osteopenia, whereas high levels of IL-18 binding protein, a natural antagonist, has been positively correlated with LS-BMAD [53]. This suggests that IL-18's effect on bone may be modulated by estrogen, which fluctuates during puberty. In a randomized control trial in healthy postmenopausal women <60 years old, administration of raloxifene, a selective estrogen receptor modulator, for 6 months showed a significant decrease in serum IL-18 concentrations [54]. In adult males with HIV virally suppressed on ART, elevated IL-18 was associated with lipodystrophy, a marker of disease progression [55]. There are no similar data among AWH.

As expected, CRP, sCD14, and TNF-α were associated with HIV status; CRP and TNF-α, both indicators of innate immune activation, and sCD14, a marker of monocyte activation, have been consistently associated with chronic inflammation and increased morbidity in HIV [56, 57]. CRP and TNF-α have been shown to stimulate RANKL expression and activate NF-κB signaling, thereby promoting osteoclast differentiation [58–61], with TNF-α further enhancing this process through upregulation of CSF1 receptor expression on osteoclast precursors [62]. sCD14 has similarly been reported to promote osteoclast differentiation, although its underlying mechanisms remain uncertain [63]. Among the general population, elevated CRP, sCD14, and TNF-α are associated with increased risk of osteoporosis and fracture [64–66]. However, among people with HIV with low bone density, it remains unclear to what extent this is driven by elevated CRP, TNF-α, or sCD14 [67, 68]. These effects were not observed in exploratory analysis of component 1, where IL-18 was the primary driver.

We found no association between component 1 and lean mass, grip strength, or jump power, contrasting with adult data where inflammation has been shown to be negatively correlated with grip strength [69]. A longitudinal study in the United States involving adults with HIV (median age, 36 years) reported decreased inflammation following ART initiation that was associated with increased muscle density [20]. Pubertal hormonal levels may mask inflammation's catabolic effects on muscle. Interestingly, among girls without HIV, component 2 was positively associated with grip strength, suggesting that signals may interact with hormones to support muscle development in AWOH. The absence of such effects in AWH may reflect disrupted immune-muscle signaling in HIV; however, this current study may be underpowered to detect differences.

There was no association between biomarkers and ART duration or viral load. It is known that ART reduces inflammation; however, levels often remain above those in uninfected individuals, likely due to viral reservoirs, microbial translocation, or coinfections [70]. In a study of Kenyan CWH between 4 and 8 years of age, the timing of ART initiation and duration of ART was shown to have distinct immune effects. Children who were treated early after diagnosis had higher naive-to-effector memory T-cell ratios, and lower levels of inflammatory biomarkers, whereas children with delayed treatment initiation had lower naive-to-effector memory T-cell ratios and higher levels of inflammatory biomarkers despite viral load suppression [71]. The lack of association observed in our study may be attributable to the participants being older, and the variability in ART duration between participants, which may have obscured any effect on biomarkers. The lack of association between biomarkers and HIV viral load in our study is consistent with the literature indicating that viral load is only weakly associated with immune activation in children [72]. This may be contributed to by a more active thymus in childhood and adolescents than in adults, facilitating more efficient replenishment of CD4^+^ T cells [73].

Other factors that may impair bone density, muscle mass, and strength, such as inadequate dietary calcium, vitamin D deficiency [74, 75], low levels of physical activity [76], and orphanhood [75], have less well-established associations on the immune biomarkers and therefore were not further investigated in this study.

The strengths of this study include the HIV-negative comparison group, data detailing HIV-related factors, and focused measurement of cytokines relevant to the musculoskeletal outcomes. Limitations are that the study may be underpowered to detect differences by HIV status and sex; causality cannot be inferred as it is a cross-sectional design; possible residual confounding from unmeasured biological or environmental factors; and absence of sex and/or growth hormone measurements. The 4 continuous variables (IL-18, CRP, sCD14, TNF-α) loaded highest onto one component and the 4 categorical variables (IL-6, IL-10, IL-17, IFN-γ) onto the other, but a sensitivity analysis using binarized variables for all 8 components produced similar results. We did not collect further data about ART adherence beyond viral load measurement. Although we performed DXA scan measurements according to recommendations [34], DXA scans may underestimate areal bone density in small skeletons (as occurs in AWH), and the reference data used were predominantly from White British children. To our knowledge, there are no equivalent reference data locally or elsewhere in Africa.

## CONCLUSIONS

This study demonstrates that perinatally acquired HIV is associated with elevated biomarkers relevant to bone density and muscle mass and strength among 8- to 16-year-olds in Zimbabwe. Higher LS-BMAD was observed among girls with HIV, driven by elevated IL-18. There was no association between biomarkers and muscle mass and strength in HIV in this population. Further investigation is warranted into in vitro mechanisms of these biomarkers, particularly IL-18, on bone density.

## Supplementary Material

ofag322_Supplementary_Data

## Supplementary Data

Supplementary materials are available at *Open Forum Infectious Diseases* online. Consisting of data provided by the authors to benefit the reader, the posted materials are not copyedited and are the sole responsibility of the authors, so questions or comments should be addressed to the corresponding author.
