# Supplementary material for: Association Between Chronic Inflammation, Bone Density, and Muscle in Peripubertal Adolescents With and Without HIV in Zimbabwe: A Cross-sectional Study
Source: Open Forum Infect Dis. 2026 May 22;13(6):ofag322. doi: 10.1093/ofid/ofag322 (PMC13229115; doi:10.1093/ofid/ofag322)
Supplement: ofag322_Supplementary_Data [file ofag322_supplementary_data.docx]

## Supplementary

Participants aged 8 to 16 years old invited to participate

**N=1197**

Government sector HIV clinics

**n=631**

Government schools nearby

**n=536**

- Not living in Harare (n=72)
- ART<2 years (n=52)
- Unaware of HIV status (n=20)
- Acute illness (n=1)

A

- Not living in Harare (n=15)
- Established HIV infection on ART >2 years (n=4)
- <8 or >16 years (n=17)

Eligible adolescents without HIV (AWOH) (n=500)

Eligible AWH (n=486)

**+ 4** identified from schools

AWH consented (n=305)

AWOH consented (n=307)

Withdrawn after consent (n=2)

**Enrolled AWH (n=303)**

**Enrolled AWOH (n=306)**

Withdrawn due to new HIV diagnosis (n=1)

- Guardian +/- child refusal (n=98)
- No guardian present (n=87)

- Letter not returned (n=151)
- No guardian present or child refused (n=42)

**Blood plasma sample (n= 270)**

**Blood plasma sample (n= 287)**

No blood sample (n=33)

- Not fasted
- Difficulty collecting sample

No blood sample (n=19)

- Not fasted
- Difficulty collecting sample

**Supplementary Figure 1: Study participants recruitment and follow-up after one year**

ART=antiretroviral therapy. AWH= Adolescents with HIV, AWOH= Adolescents without HIV, DXA=dual-energy X-ray absorptiometry.

Alt text: Flow chart of the study participants recruited. Each step denoted in a box. AWH with a blood plasma sample was 270; and those without HIV with a blood sample was 287

**Supplementary Materials 1:**

##### Missing data characteristics

There were 28 (9.2%) boys and 24 (7.8%) girls who did not have a plasma sample. Boys with a missing plasma sample were of higher socio-economic status and did less physical activity than boys who had a plasma sample. Of the 557 participants, there were 6 (2.2%) boys and 10 (3.5%) girls missing LS-BMAD Z-score data; 7 (2.5%) boys and 10 girls (3.5%) missing TBLH-BMC^LBM^ Z-score data; 2 (0.7%) boys and 3 girls (1.1%) missing long jump data; and 7 boys (2.5%) and 10 girls (3.5%) missing lean mass data. No data was missing for grip strength. Males with at least one missing outcome were on average 2 years older than boys with complete data, and at higher Tanner stage (Supplementary Table 2). Girls with at least one missing outcome had higher vitamin D consumption (Supplementary Table 2). Other characteristics between those with at least one missing outcome and those with complete entries were similar (Supplementary Table 2).

##### Biomarker data

IL-18 and sCD14 had no out-of-range values. There were 19 (3.4%) samples where TNFα was below the limit-of-detection; the lowest measured standard curve value was used. There was one sample where CRP was below limit-of-detection and eight samples above the limit-of-detection, where the lowest and highest standard curve value were used, respectively. There were 144 (25.9%) samples for IL-6; 364 (65.4%) samples for IL-10; 271 (48.7%) samples for IL-17 and 254 (45.6%) samples for IFNγ where measurements were below the limit-of-detection.

Median biomarker levels were increased between AWH and AWOH across both sexes for IL-18, CRP, sCD14 and TNFα (Supplementary Table 3). IL-6 measurements were more often detectable among boys with HIV compared to boys without HIV. IL-10 measurements were more often detectable among both boys and girls with HIV compared to those without (Supplementary Table 3). When dimensionally reduced, the first two components together explained 44.2% of the total variance, with component 1 accounting for 26.5% and component 2 for 17.7% (Supplementary Figure 2a). Component 1 was primarily influenced by IL-18, TNFα, CRP, sCD14. Component 2 captured variations in IFNγ, IL-17, IL-6 and IL-10 (Supplementary Figure 2b).

**Supplementary Table 1: Baseline characteristics comparing study participants with a blood plasma sample compared to those without**

|  | | **BOYS**  **N=303** | | | **GIRLS**  **N= 306** | | |
| --- | --- | --- | --- | --- | --- | --- | --- |
|  |  | **Sample available**  **n=275** | **Missing sample**  **n=28** | **p-value** | **Sample available**  **n=282** | **Missing sample**  **n= 24** | **p-value** |
| **Sociodemographic characteristics** | Age years, mean (SD) | 12.5 (2.5) | 12.0 (2.3) | 0.253 | 12.7 (2.5) | 12.8 (2.5) | 0.766 |
|  | Socioeconomic status, n(%) |  |  |  |  |  |  |
|  | Tertile 1 (low) , n(%) | 87 (31.6) | 5 (17.9) | 0.309 | 104 (36.9) | 7 (29.17) | 0.730 |
|  | Tertile 2 (middle) , n(%) | 93 (33.8) | 12 (42.9) |  | 89 (31.6) | 9 (37.5) |  |
|  | Tertile 3 (high) , n(%) | 95 (34.5) | 11 (39.3) |  | 89 (31.6) | 8 (33.3) |  |
|  | Orphanhood (one or both parents)* | 64 (23.3) | 7 (25.0) | 1.000 | 67 (23.8) | 5 (20.8) | 0.889 |
| **Pubertal stage^#^** | Tanner I, n(%) | 92 (34.6) | 10 (38.5) | 0.592 | 77 (27.8) | 8 (36.4) | 0.051 |
|  | Tanner II, n(%) | 64 (24.1) | 9 (34.6) |  | 53 (19.1) | 2 (9.1) |  |
|  | Tanner III, n(%) | 43 (16.2) | 3 (11.5) |  | 54 (19.5) | 8 (36.4) |  |
|  | Tanner IV, n(%) | 59 (22.2) | 3 (11.5) |  | 73 (26.4) | 1 (4.5) |  |
|  | Tanner V, n(%) | 8 (3.1) | 1 (3.8) |  | 20 (7.2) | 3 (13.6) |  |
| **Lifestyle factors** | Physical activity level, n(%) |  |  |  |  |  |  |
|  | Low, <600 MET mins/week | 107 (38.9) | 15 (53.6) | 0.289 | 130 (46.1) | 10 (41.7) | 0.835 |
|  | Moderate, 600-3000 MET mins/week | 78 (28.3) | 7 (25.0) |  | 74 (26.2) | 6 (25.0) |  |
|  | High, >3000 MET mins/week | 90 (32.7) | 6 (21.4) |  | 78 (27.7) | 8 (33.3) |  |
|  | Daily dietary vitamin D, n(%) |  |  |  |  |  |  |
|  | Very low, <4.0 μg/day | 13 (4.7) | 2 (7.1) | 0.553 | 14 (5) | 3 (12.5) | 0.185 |
|  | Low, 4.0-5.99 μg/day | 156 (56.7) | 13 (46.4) |  | 161 (57.1) | 15 (62.5) |  |
|  | Moderate, 6.0-8.0 μg/day | 106 (38.5) | 13 (46.4) |  | 107 (37.9) | 6 (25) |  |
|  | History of tuberculosis^, n(%) | 28 (89.8) | 25 (89.3) | 1.000 | 18 (6.4) | 2 (8.3) | 1.000 |
| MET= resting metabolic rate. TDF= tenofovir disoproxil fumarate. LS-BMAD= lumbar spine bone mineral apparent density. TBLH-BMC^LBM^= total body- less head bone mineral content for lean body mass. ART= antiretroviral treatment.  Missing datapoints:  *Orphanhood (boys: one from missing, eight from sample; girls: none from missing, seven from sample)  ^History of tuberculosis (boys: none; girls: none from missing, two from sample)  ^#^Pubertal stage (boys: 9 from sample, two from missing; girls: 5 from sample, two from missing) | | | | | | | |

**Supplementary Table 2: Baseline characteristics comparing participants with all musculoskeletal outcome compared to those with at least one missing musculoskeletal outcome**

|  | | **BOYS**  **N=275** | | | **GIRLS**  **N= 282** | | |
| --- | --- | --- | --- | --- | --- | --- | --- |
|  |  | **Complete outcomes**  **n=266** | **Missing at ≥1 MS outcome**  **n=13** | **p-value** | **Complete outcomes**  **n=269** | **Missing at ≥1 MS outcome**  **n=13** | **p-value** |
| **Sociodemographic characteristics** | Age years, mean (SD) | 12.4 (2.5) | 14.4 (1.9) | 0.013 | 12.5 (2.5) | 12.9 (2.7) | 0.625 |
|  | Socioeconomic status, n(%) |  |  |  |  |  |  |
|  | Tertile 1 (low) , n(%) | 84 (31.6) | 3 (33.3) | 0.993 | 99 (36.8) | 5 (38.5) | 0.357 |
|  | Tertile 2 (middle) , n(%) | 90 (33.9) | 3 (33.3) |  | 83 (30.9) | 6 (46.2) |  |
|  | Tertile 3 (high) , n(%) | 92 (34.6) | 3 (33.3) |  | 87 (32.3) | 2 (15.4) |  |
|  | Orphanhood (one or both parents)* , n(%) | 63 (23.7) | 1 (11.1) | 0.725 | 62 (23.0) | 5 (38.5) | 0.378 |
| **Pubertal stage** | Tanner I, n(%) | 91 (35.4) | 1 (11.1) | 0.406 | 74 (28.0) | 3 (23.1) | 0.885 |
|  | Tanner II, n(%) | 62 (24.1) | 2 (22.2) |  | 51 (19.3) | 2 (15.4) |  |
|  | Tanner III, n(%) | 40 (15.6) | 3 (33.3) |  | 50 (18.9) | 4 (30.8) |  |
|  | Tanner IV, n(%) | 56 (21.8) | 3 (33.3) |  | 70 (26.5) | 3 (23.1) |  |
|  | Tanner V, n(%) | 8 (3.11) | 0 (0) |  | 19 (7.2) | 1 (7.7) |  |
| **Lifestyle factors** | Physical activity level, n(%) |  |  |  |  |  |  |
|  | Low, <600 MET mins/week | 103 (38.7) | 4 (44.4) | 0.906 | 124 (46.1) | 6 (46.2) | 0.951 |
|  | Moderate, 600-3000 MET mins/week | 76 (28.6) | 2 (22.2) |  | 71 (26.4) | 3 (23.1) |  |
|  | High, >3000 MET mins/week | 87 (32.7) | 3 (33.3) |  | 74 (27.5) | 4 (30.8) |  |
|  | Daily dietary vitamin D, n(%) |  |  |  |  |  |  |
|  | Very low, <4.0 μg/day | 13 (4.9) | 0 (0) | 0.500 | 14 (5.2) | 0 (0) | 0.392 |
|  | Low, 4.0-5.99 μg/day | 152 (57.1) | 4 (44.4) |  | 155 (57.6) | 6 (46.1) |  |
|  | Moderate, 6.0-8.0 μg/day | 101 (38) | 5 (55.6) |  | 100 (37.2) | 7 (53.9) |  |
|  | History of tuberculosis^, n(%) | 26 (9.8) | 2 (2.22) | 0.513 | 17 (6.3) | 1 (7.7) | 1.00 |
| MET= resting metabolic rate. TDF= tenofovir disoproxil fumarate. LS-BMAD= lumbar spine bone mineral apparent density. TBLH-BMC^LBM^= total body- less head bone mineral content for lean body mass. ART= antiretroviral treatment. MS= musculoskeletal (bone density, muscle mass and strength)  Missing datapoints:  *Orphanhood (boys: seven from complete, one from incomplete; girls: seven from complete, none from incomplete)  ^History of tuberculosis (boys: none; girls: seven from complete, none from incomplete) | | | | | | | |

**Supplementary Table 3: Distribution of biomarkers**

|  | **Girls (n=282)** | | **Boys (n=275)** | |
| --- | --- | --- | --- | --- |
|  | **Without HIV**  **Median (IQR)**  **N= 148** | **With HIV**  **Median (IQR)**  **N=134** | **Without HIV**  **Median (IQR)**  **N=139** | **With HIV**  **Median (IQR)**  **N=136** |
| **IL-18 (pg/ml)** | 196.62 (123.97; 288.39) | 228.67 (164.97; 365.01) | 205.89 (143.67; 289.40) | 224.76 (143.56; 351.88) |
| **CRP (mg/dl)** | 0.023 (0.007; 0.076) | 0.046 (0.013; 0.177) | 0.014 (0.005; 0.065) | 0.024 (0.009; 0.132) |
| **sCD14 (ng/ml)** | 1200.1 (820.40; 1557.60) | 1600.40 (1127.50; 2257.50) | 1083.70 (791.15; 1506.75) | 1541.10 (930.90; 2152.50) |
| **TNFα (pg/ml)** | 2.99 (1.81; 4.72) | 4.15 (2.79; 6.56) | 3.73 (2.64; 5.32) | 4.92 (3.13; 7.33) |
|  | **Without HIV.**  **Detectable measurement* n(%)**  **N= 148** | **With HIV.**  **Detectable measurement* n(%)**  **N=134** | **Without HIV.**  **Detectable measurement* n(%)**  **N=139** | **With HIV.**  **Detectable measurement* n(%)**  **N=136** |
| **IL-6** | 89 (64.0) | 109 (80.15) | 114 (77.03) | 101 (75.37) |
| **IL-10** | 42 (30.22) | 56 (41.18) | 37 (25.0) | 58 (41.79) |
| **IL-17** | 68 (48.92) | 78 (57.35) | 73 (49.32) | 66 (48.53) |
| **IFNγ** | 77 (55.40) | 76 (55.88) | 80 (54.05) | 71 (52.21) |
| * Detectable means within the standard curve. All undetectable values were below the lower limit of detection.  CRP= C-reactive protein. TNFα= tumour necrosis factor alpha. IL= interleukin. IFNγ= interferon gamma. | | | | |

**Supplementary Figure 2a and 2b: Scree plot and plot of contributing variables to respective dimensions (i.e. components)**

Alt text: Scree plot on the left showing the percentage variance explained by each dimension/component. Component 1: 27%, 2: 17.9%; 3:15.1%; 4: 11.9%; 5:9.9%. Scatter plot on the left showing the variable contributions to a factorial analysis of mixed data (FAMD). Dimension 1 separates markers TNFα, CRP, sCD14 and IL-18 to the right, while dimension/component 2 separates IL-6, IL-10, IL-17 and IFNγ upward.


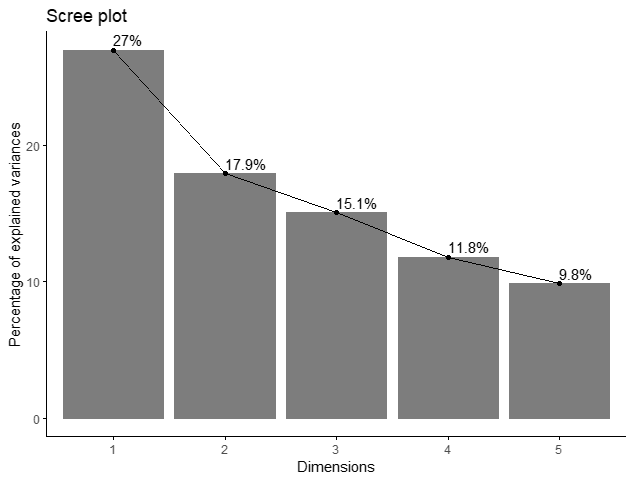

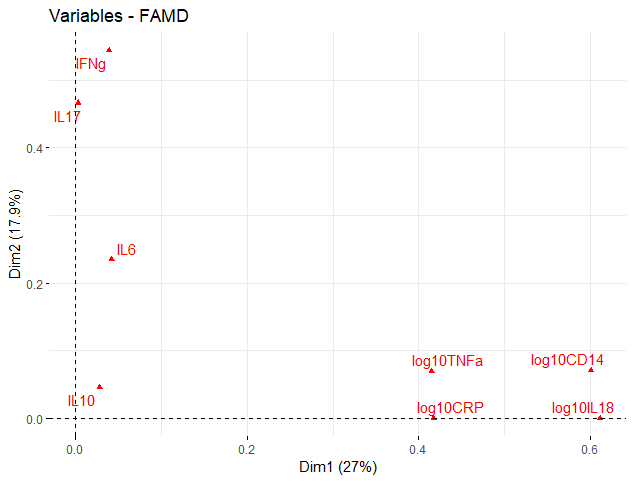


**Supplementary Table 4: A linear regression model (95% confidence interval) of risk factors associated with component 1 biomarkers among adolescents with HIV**

|  | | **GIRLS (n=134)** | | | | |  | **BOYS (n=136)** | | | | |  |
| --- | --- | --- | --- | --- | --- | --- | --- | --- | --- | --- | --- | --- | --- |
|  |  | **Univariable**  **(β coefficient, 95% CI)** | **p- value** | **Test for trend)** | **Multivariable^1^**  **(β coefficient, 95% CI)** | **p- value** | **Test for trend** | **Univariable**  **(β coefficient, 95% CI)** | **p- value** | **Test for trend** | **Multivariable^1^**  **(β coefficient, 95% CI)** | **p- value** | **Test for trend** |
| **Age (years)** | | -0.02 (-0.12 to 0.07) | 0.618 |  | 0.08 (-0.10; 0.26) | 0.375 |  | -0.07 (-0.17 to 0.04) | 0.198 |  | -0.11 (-0.28; 0.06) | 0.194 |  |
| **Pubertal Stage** | **I** | - | - | 0.630 | - | - | 0.191 | - | - | 0.724 | - | - | 0.355 |
|  | **II** | -0.26 (-1.02 to 0.50) | 0.496 |  | -0.44 (-1.33; 0.45) | 0.331 |  | -0.21 (-0.87 to 0.45) | 0.527 |  | 0.07 (-0.68; 0.81) | 0.860 |  |
|  | **III** | -0.01 (-0.70 to 0.67) | 0.968 |  | -0.26 (-1.20; 0.68) | 0.587 |  | -0.59 (-1.39 to 0.22) | 0.154 |  | -0.14 (-1.17; 0.88) | 0.785 |  |
|  | **IV** | -0.67 (-1.36 to 0.02) | 0.058 |  | -1.16 (-2.29; -0.03) | 0.045 |  | -0.39 (-1.20 to 0.42) | 0.342 |  | 0.07 (-1.10; 1.23) | 0.911 |  |
|  | **V** | -0.10 (-1.27 to 1.07) | 0.866 |  | -0.86 (-2.49; 0.77) | 0.298 |  | 0.36 (-1.08 to 1.80) | 0.624 |  | 0.96 (-0.80; 2.73) | 0.283 |  |
| **ART duration (years)** | | -0.09 (-0.19 to 0.01 | 0.084 |  | -0.08 (-0.18; 0.02) | 0.114 |  | -0.01 (-0.10 to 0.09) | 0.889 |  | -0.02 (-0.12; 0.09) | 0.750 |  |
| **Viral Load**  **(copies/ml)** | **<=50** | - | - | 0.177 | - | - | 0.776 | - | - | 0.101 | - | - | 0.226 |
|  | **51-999** | 0.24 (-0.43 to 0.90) | 0.480 |  | -0.01 (-0.69; 0.68) | 0.983 |  | -0.27 (-0.91 to 0.36) | 0.400 |  | -0.30 (-0.94; 0.35) | 0.368 |  |
|  | **>=1000** | 0.56 (-0.08 to 1.20) | 0.083 |  | 0.62 (-0.03; 1.27) | 0.062 |  | 0.41 (-0.27 to 1.09) | 0.233 |  | 0.56 (-0.18; 1.29) | 0.138 |  |
| 1. Multivariable linear regression model for the association between component 1 biomarkers and ART duration and viral load, adjusted for age, pubertal stage and fat mass   β coefficient is the increase or decrease in component 1 biomarkers per one-unit increase in the risk factor. TBLH= total body-less head. ART= antiretroviral treatment. CI= Confidence interval. | | | | | | | | | | | | |  |


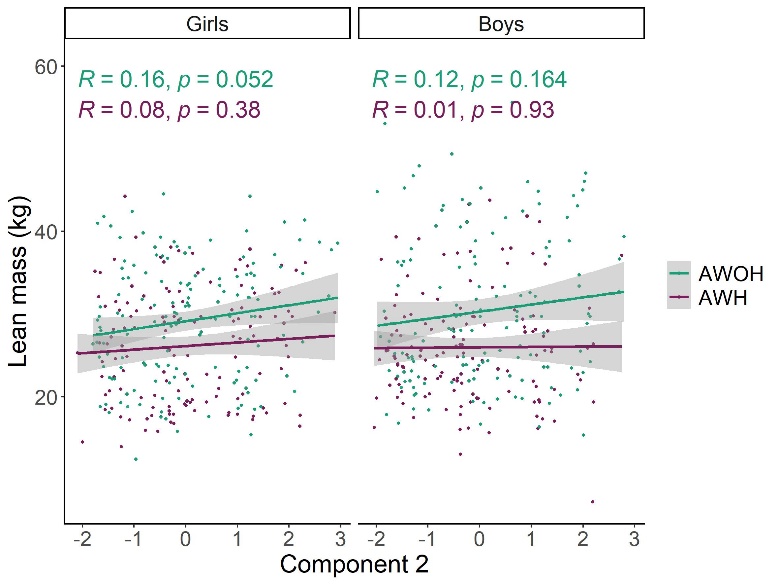

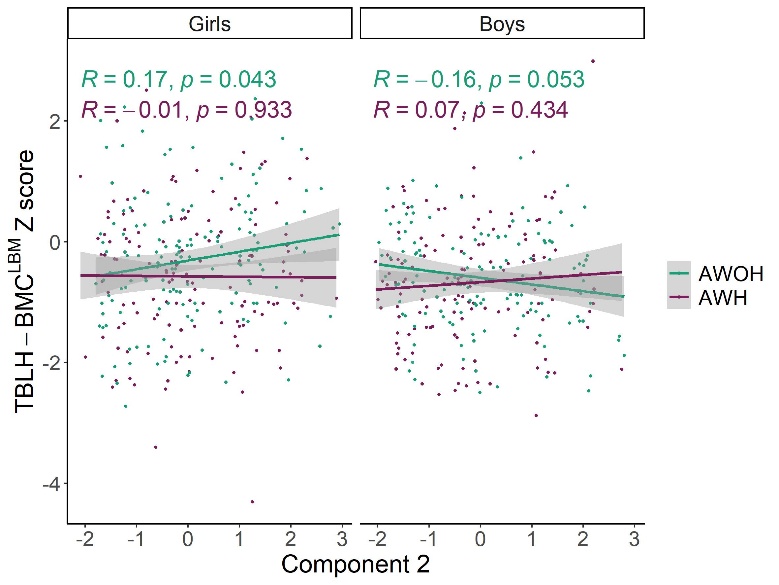

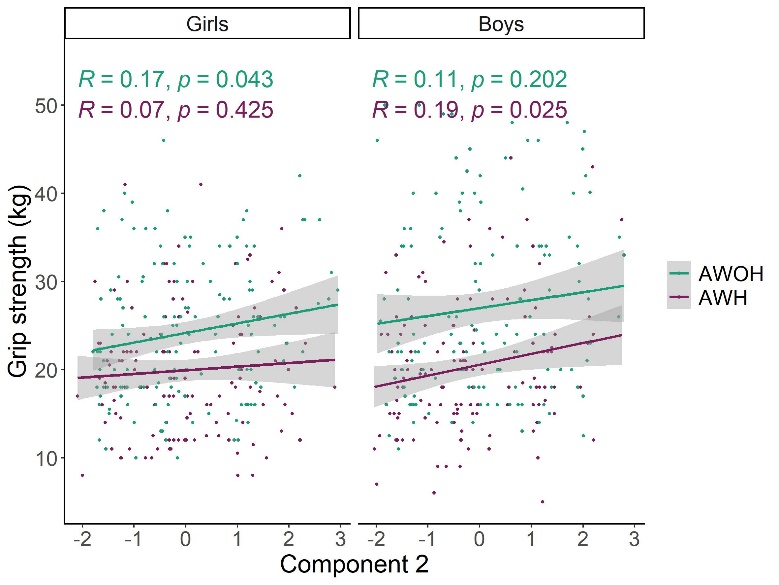

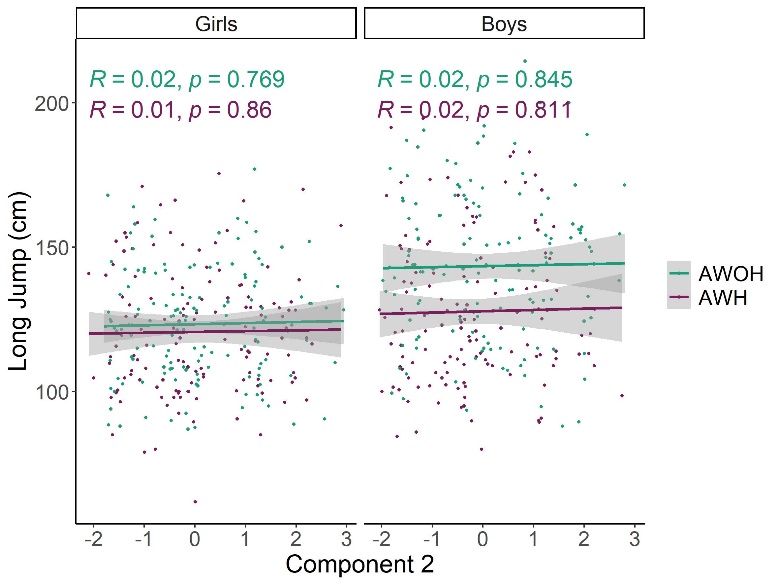

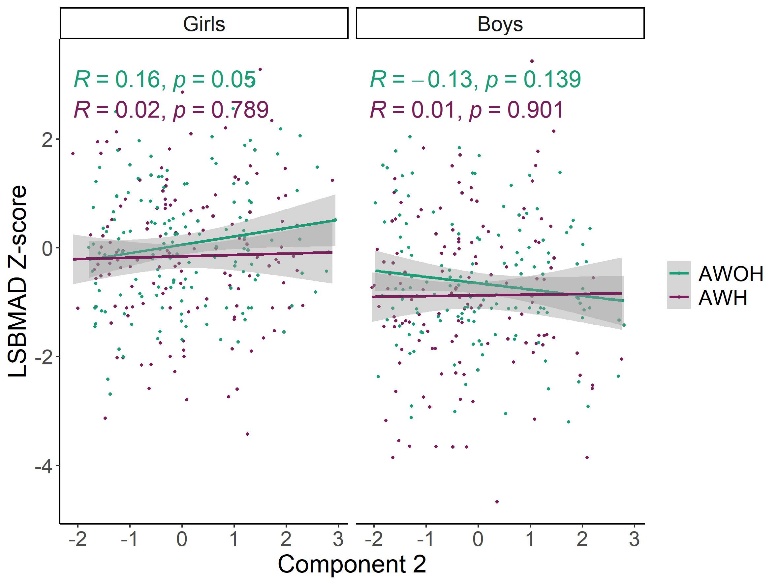


**(a)**

**(b)**

**(c)**

**(d)**

**(e)**

**Supplementary Figure 3:** Correlations between component 1 biomarkers and musculoskeletal outcomes

AWH= Adolescents with HIV. AWOH= Adolescents without HIV. LS-BMAD= lumbar spine bone mineral apparent density. TBLH-BMC^LBM^= total body-less head bone mineral content for lean body mass. Unadjusted data are shown. Pearson’s correlation coefficient (R) shown with p-value. Error bars (in grey) indicate 95% confidence intervals. Data presented in teal represent AWOH and purple represents AWH.

Alt Text (Supplementary Figure 3): Scatter plots to compare bone density and muscle outcomes (lean mass, grip strength, jump power) for eigenvalues of component 2 in girls (left panel) and boys (right panel). Lines show trends for adolescents without HIV in teal and with HIV in purple.

|  | **LSBMAD Z-score** | | | | **TBLH-BMC**^LBM^ **Z-score** | | | |
| --- | --- | --- | --- | --- | --- | --- | --- | --- |
|  | **Univariable coefficient^1^ (95% CI)** | **p- value** | **Multivariable coefficient^1^ (95% CI)** | **p-value** | **Univariable coefficient^1^ (95% CI)** | **p-value** | **Multivariable coefficient^1^ (95% CI)** | **p-value** |
| **GIRLS (n=134)** | | | | | | | | |
| Component 1 | -0.03 (-0.16; 0.11) | 0.698 | -0.04 (-0.18; 0.09) | 0.528 | 0.05 (-0.08; 0.17) | 0.454 | 0.02 (-0.10; 0.14) | 0.704 |
| Component 2 | 0.15 (0.00; 0.31) | 0.050 | 0.11 (-0.04; 0.26) | 0.148 | 0.15 (0.00; 0.29) | 0.043 | 0.11 (-0.02; 0.25) | 0.089 |
| **BOYS (n=136)** | | | | | | | | |
| Component 1 | 0.05 (-0.10; 0.20) | 0.486 | 0.00 (-0.15; 0.15) | 0.984 | -0.01 (-0.12; 0.10) | 0.838 | -0.02 (-0.13; 0.10) | 0.761 |
| Component 2 | -0.11 (-0.27; 0.04) | 0.139 | -0.06 (-0.21; 0.10) | 0.480 | -0.11 (-0.22; 0.00) | 0.053 | -0.13 (-0.25; -0.02) | 0.026 |
| 1. Adjusted for age (years), pubertal stage (5 levels) and fat mass (kg)   β coefficient is the increase or decrease in component 1 or 2 biomarkers per one-unit increase in the bone density Z-score.  CI= confidence interval. | | | | | | | | |

**Supplementary Table 5: Linear regression coefficients (95% confidence interval) for the association between biomarker components and bone density among adolescents without HIV**

**Supplementary Table 6: Linear regression coefficients (95% confidence interval) for the association between biomarkers and bone density outcomes among adolescents with HIV separated by Pubertal (Tanner) stage**

|  | **LS-BMAD Z-score** | | | | **TBLH-BMC**^LBM^ **Z-score** | | | |
| --- | --- | --- | --- | --- | --- | --- | --- | --- |
|  | **Univariable coefficient**  **(95% CI)** | **p-value** | **Multivariable coefficient^1^**  **(95% CI)** | **p-value** | **Univariable coefficient**  **(95% CI)** | **p-value** | **Multivariable coefficient^1^**  **(95% CI)** | **p-value** |
| **Tanner I to II** | | | | | | | | |
| **Girls (n=75)** | | | | | | | | |
| Component 1 | 0.08 (-0.14; 0.30) | 0.453 | 0.11 (-0.10; 0.32) | 0.293 | 0.06 (-0.11; 0.23) | 0.454 | 0.06 (-0.10; 0.23) | 0.453 |
| Component 2 | 0.01 (-0.28; 0.30) | 0.963 | 0.00 (-0.28; 0.28) | 0.987 | 0.03 (-0.19; 0.25) | 0.774 | -0.00 (-0.22; 0.22) | 0.968 |
| **Boys (n=91)** | | | | | | | | |
| Component 1 | -0.02 ( -0.19; 0.15) | 0.153 | -0.05 (-0.21; 0.12) | 0.570 | -0.04 (-0.17; 0.09) | 0.512 | -0.05 (-0.18; 0.08) | 0.435 |
| Component 2 | 0.14 (-0.12; 0.40) | 0.281 | 0.07 (-0.19; 0.34) | 0.579 | -0.04 (-0.23; 0.15) | 0.688 | -0.09 (-0.30; 0.12) | 0.401 |
| **Tanner III to V** | | | | | | | | |
| **Girls (n=59)** | | | | | | | | |
| Component 1 | 0.11 (-0.09; 0.31) | 0.273 | 0.21 (0.03; 0.40) | 0.024 | 0.06 (-0.16; 0.27) | 0.595 | 0.17 (-0.03; 0.38) | 0.100 |
| Component 2 | 0.04 (-0.19; 0.27) | 0.742 | 0.09 (-0.13; 0.31) | 0.430 | -0.06 (-0.31; 0.19) | 0.635 | -0.09 (-0.33; 0.15) | 0.464 |
| **Boys (n=45)** | | | | | | | | |
| Component 1 | -0.20 (-0.51; 0.12) | 0.216 | -0.16 (-0.41; 0.08) | 0.182 | 0.05 (-0.20; 0.29) | 0.712 | 0.05 (-0.21; 0.31) | 0.703 |
| Component 2 | -0.10 (-0.44; 0.25) | 0.594 | -0.01 (-0.28; 0.27) | 0.963 | 0.25 (0.00; 0.51) | 0.048 | 0.31 (0.04; 0.58) | 0.024 |
| 1. Adjusted for age (years), pubertal stage (5 levels) and fat mass (kg)   β coefficient is the increase or decrease in component 1 or 2 biomarkers per one-unit increase in the bone density Z-score.  CI= confidence interval. | | | | | | | | |

**Supplementary Table 7: Linear regression coefficients (95% confidence interval) for the association between biomarker components and muscle outcomes among adolescents with HIV**

|  | **Lean mass (kg)** | | | | **Grip strength (kg)** | | | | **Jump power (cm)** | | | |
| --- | --- | --- | --- | --- | --- | --- | --- | --- | --- | --- | --- | --- |
|  | **Unadjusted**  **(β coefficient, 95% CI)** | **p value** | **Adjusted**  **(β coefficient, 95% CI) ^1^** | **p value** | **Unadjusted**  **(β coefficient, 95% CI)** | **p value** | **Adjusted**  **(β coefficient, 95% CI) ^1^** | **p value** | **Unadjusted**  **(β coefficient, 95% CI)** | **p value** | **Adjusted**  **(β coefficient, 95% CI) ^1^** | **p value** |
| **GIRLS (n=134)** | | | | | | | | | | | | |
| Component 1 | -0.54 (-1.33; 0.25) | 0.177 | -0.18 (-0.63; 0.28) | 0.438 | -0.16 (-0.99; 0.67) | 0.710 | 0.03 (-0.53; 0.60) | 0.904 | -0.25 (-2.76; 2.28) | 0.845 | -0.66 (-3.00; 1.68) | 0.578 |
| Component 2 | 0.43 (-0.54; 1.41) | 0.380 | 0.22 (-0.33; 0.77) | 0.431 | 0.41 (-0.61; 1.44) | 0.425 | 0.19 (-0.51; 0.88) | 0.594 | 0.28 (-2.84; 3.40) | 0.860 | -0.05 (-2.91; 2.80) | 0.971 |
| **BOYS (n=136)** | | | | | | | | | | | | |
| Component 1 | -0.55 (-1.26; 0.17) | 0.132 | -0.19 (-0.68; 0.30) | 0.439 | -0.43 (-1.23; 0.36) | 0.291 | -0.02 (-0.57; 0.53) | 0.940 | -1.29 (-4.04; 1.47) | 0.357 | -0.21 (-2.51; 2.10) | 0.859 |
| Component 2 | 0.04 (-0.94; 1.02) | 0.930 | -0.65 (-1.33; 0.04) | 0.063 | 1.23 (0.15; 2.30) | 0.025 | 0.64 (-0.12; 1.41) | 0.100 | 0.455 (-3.30; 4.21) | 0.811 | -1.92 (-5.15; 1.33) | 0.246 |
| 1. Adjusted for age (years), pubertal stage (5 stages) and fat mass (kg)   β coefficient is the increase or decrease in component 1 or 2 biomarkers per one-unit increase in the muscle outcome.  CI= confidence interval. | | | | | | | | | | | | |

**Supplementary Table 8: Linear regression coefficients (95% confidence interval) for the association between muscle outcomes and biomarkers among adolescents without HIV**

|  | **Lean mass (kg)** | | | | **Grip strength (kg)** | | | | **Jump power (cm)** | | | |
| --- | --- | --- | --- | --- | --- | --- | --- | --- | --- | --- | --- | --- |
|  | **Univariable coefficient**  **(95% CI)** | **p value** | **Multivariable coefficient^1^**  **(95% CI)** | **p value** | **Univariable coefficient**  **(95% CI)** | **p value** | **Multivariable coefficient^1^**  **(95% CI)** | **p value** | **Univariable coefficient**  **(95% CI)** | **p value** | **Multivariable coefficient^1^**  **(95% CI)** | **p value** |
| **GIRLS (n=134)** | | | | | | | | | | | | |
| Component 1 | -0.70 (-1.56; 0.17) | 0.112 | -0.17 (-0.65; 0.32) | 0.498 | -0.37 (-1.32; 0.59) | 0.447 | 0.15 (-0.45; 0.75) | 0.624 | -0.07 (-3.0; 1.58) | 0.545 | 0.35 (-1.88; 2.58) | 0.757 |
| Component 2 | 0.96 (-0.01; 1.93) | 0.052 | 0.35 (-0.18; 0.87) | 0.195 | 1.09 (0.03; 2.15) | 0.043 | 0.57 (-0.08; 1.21) | 0.087 | 0.38 (-2.19; 2.96) | 0.769 | 0.47 (-1.98; 2.93) | 0.705 |
| **BOYS (n=136)** | | | | | | | | | | | | |
| Component 1 | -1.32 (-2.46; -0.17) | 0.025 | -0.39 (-1.01; 0.24) | 0.227 | -1.18 (-2.49; 0.14) | 0.079 | 0.05 (-0.66; 0.77) | 0.882 | -2.43 (-5.72; 0.87) | 0.147 | -0.35 (-2.91; 2.21) | 0.785 |
| Component 2 | 0.86 (-0.36; 2.08) | 0.164 | 0.15 (-0.51; 0.82) | 0.647 | 0.90 (-0.49; 2.29) | 0.202 | -0.03 (-0.78; 0.73) | 0.935 | 0.34 (-3.14; 3.83) | 0.845 | -0.40 (-3.11; 2.30) | 0.769 |
| 1. Adjusted for age (years), pubertal stage (5 levels) and fat mass (kg)   β coefficient is the increase or decrease in component 1 or 2 biomarkers per one-unit increase in the muscle outcome.  CI= confidence interval. | | | | | | | | | | | | |
